# COVID-19 Testing and Vaccine Hesitancy in Latinx Farm-Working Communities in The Eastern Coachella Valley

**DOI:** 10.21203/rs.3.rs-587686/v1

**Published:** 2021-06-25

**Authors:** Daniel Gehlbach, Evelyn Vázquez, Gabriela Ortiz, Erica Li, Cintya Beltrán Sánchez, Sonia Rodríguez, María Pozar, Ann Marie Cheney

**Affiliations:** University of California Riverside School of Medicine; University of California Riverside School of Medicine; University of California, Riverside; University of California Riverside School of Medicine; University of California Riverside School of Medicine; University of California Riverside Center for Health Disparities Research; University of California Riverside Center for Health Disparities Research; University of California Riverside School of Medicine

**Keywords:** COVID-19, health disparities, immigrant, migrant, Latinx health, community based participatory research, Indigenous Mexicans

## Abstract

**Background:**

A novel coronavirus, SARS-CoV-2 (known as COVID-19), spread rapidly around the world, affecting all and creating an ongoing global pandemic. In the United States, Latinx, African American, and Indigenous populations across the country have been disproportionately affected by COVID-19 cases and death rates. An examination of the perceptions and beliefs about the spread of the virus, COVID-19 testing, and vaccination amongst racial/ethnic minority groups is needed in order to alleviate the widespread disparity in new cases and deaths.

**Methods:**

From November to December 2020 the research team conducted focus groups with members of Latinx farm-working communities in the Eastern Coachella Valley, located in the inland southern California desert region. A total of seven focus groups, six in Spanish and one in Purepecha, with a total of 55 participants were conducted. Topics covered include knowledge of the coronavirus, COVID-19 testing and vaccination.

**Results:**

Using theme identification techniques, the findings identify structural factors that underly perceptions held by immigrant, migrant, and indigenous Latinx community members about COVID-19, which, in turn, shape attitudes and behaviors related to COVID-19 testing and vaccination. Common themes that emerged across focus groups include misinformation, lack of trust in institutions, and insecurity around employment and residency.

**Conclusions:**

This racial/ethnic minority population is structurally vulnerable to historical and present-day inequalities that put them at increased risk of COVID-19 exposure, morbidity, and mortality. Findings from the focus groups indicate a significant need for interventions that decrease structural vulnerabilities by addressing issues of (dis)trust in government and public health among this population.

## Introduction

Across the United States (US), African American/Black, Latinx, and Indigenous communities are disproportionately at risk for contracting SARS-CoV-2, a novel coronavirus that causes the COVID-19 respiratory disease.[1] African American/Black and Indigenous populations are 2.3 times more likely to die from COVID-19 than White populations, [2] a widening racial-ethnic disparity in mortality. In the US and across the globe, the pandemic has magnified the historically rooted systemic and social factors shaping health disparities among marginalized communities of color.[3, 4] As Frank Snowden, professor of history of medicine at Yale, pointed out, “epidemic disease are not random events that afflict societies capriciously and without warning… every society produces its own specific vulnerabilities.”[5] The coronavirus has moved and continues to move along the “fault lines created by poverty and inequality.”[6] To mitigate the impacts of coronavirus on vulnerable communities, partnered research engaging community leaders and trusted members, and acknowledging the histories of inequality and mistreatment that have shaped health disparities, is needed.

### Social And Structural Determinants Of Health

Structural inequalities and social determinants of health place disadvantaged individuals and populations at greater risk for contagion and death.[7, 8] The public health field has traditionally focused on social determinants of health (SDOH), highlighting how the conditions into which individuals are “born, grow, live, work, and age-are significant drivers of disease risk and susceptibility within clinical care and public health systems.”[9] Yet, as social medicine and related fields have argued for some time, structural level factors underlie the development and distribution of SDOH and have historically set up and put into motion inequities in health.[9, 10] Historical factors such as colonization, forced repatriation, land loss, and slavery render certain racial-ethnic groups more susceptible to the harmful and detrimental outcomes of the power imbalances embedded within institutions and social life.[11–14] These inequities are seen in higher rates of cardiovascular disease, diabetes, respiratory disease, hypertension, cancer, and infectious disease in marginalized communities of color.[15]

In the context of COVID-19, social and structural determinants of health, including sociocultural, economic, political, and environmental factors coupled with histories of abuse, exploitation, and oppression, increase risk for COVID-19 transmission and death.[3, 16] Studies show income inequality (e.g., low wages, essential labor), housing and neighborhood density,[17] geographic location (e.g., rural vs. urban),[18, 19] xenophobic and racialized climates,[20, 21] and distrust in public health and the government[22] contribute to health disparities and higher rates of infection, hospitalizations, and deaths in underserved and vulnerable communities.[16]

Therefore, this study explored the social (present-day) and structural (historical) factors shaping knowledge and perceptions of the coronavirus and COVID-19, as well as how SDOH and structural factors combined to influence attitudes and behaviors around COVID-19 testing services and future vaccine uptake. We examined this among Latinx immigrant communities in the inland southern California desert region.

## Methods

### Project Overview

This study was carried out from September 2020 to January 2021. We used principles of community based participatory research (CBPR), an approach that draws on the strengths of diverse partners, shares resources, and fosters shared decision making and knowledge creation.[23] Aligning with this approach, we convened a community advisory board that met monthly to oversee the project, facilitate relationship building, and offer advice. The overall project included three aims: 1) support the delivery of COVID-19 testing services, 2) broadly disseminate COVID-19 public health information, and 3) conduct research on COVID-19 perceptions, that were carried out with a focus on Latinx immigrants in rural agricultural communities. Prior to the start of data collection, we obtained ethical approval for the study from the University of California Riverside’s Institutional Review Board.

### Study Setting

Our study focused on the effects of the coronavirus pandemic amongst Latinx immigrants in the desert region of inland southern California. Our research was carried out in Riverside County, an area of California disproportionately impacted by the pandemic. At the time of the study, Riverside County had the second highest number of confirmed cases and deaths in the state.[24, 25] There are over 2 million Latinos in Riverside County, a majority minority population that outnumbers all other racial and ethnic groups in the region.[26–28] Most Latinos are of Mexican origin, with smaller numbers of Puerto Ricans, Salvadorans, and Guatemalans, and Indigenous Nations.[29] Latinos in this region suffer health disparities due to low income and education, limited English proficiency, and undocumented status.[30–32] Unsurprisingly, the pandemic has severely impacted this population in the region: At research inception in fall 2020, which aligned with wave two spread of the coronavirus in California, county level data indicated the Latinx population accounted for 57% of COVID-19 cases and 46% of deaths in both Riverside County.[24, 25, 33, 34]

Our study[35] focused on engaging immigrant, migrant, and indigenous Latinx in rural agricultural communities in the inland desert region of Riverside County, specifically the eastern part of the Coachella Valley. The Coachella Valley, a 45-mile-long valley encompassing nine cities and rural agricultural communities, is an area of particular racial-ethnic disparity. This area is home to several vulnerable communities including the unincorporated rural communities of the Eastern Coachella Valley (ECV): Mecca, North Shore, Oasis, and Thermal, home to many migrant and immigrant Latinx farm-working population living below the poverty line and working in the nearby agricultural fields. This region is also home to a large Purepecha community, an indigenous Mexican population from the state of Michoacan. [30] At the start of the pandemic this region was identified as a hotspot, with some reports indicating a COVID-19 infection rate in the ECV 5 times higher than other Coachella Valley communities.[36]

During the time of our study, these communities (Mecca, North Shore, Oasis, and Thermal) consistently reported the highest rates of COVID-19 cases per 1,000 residents in the Coachella Valley. For instance, in September 2020 Thermal reported >130 cases/1,000 residents, which increased to > 250 cases/1,000 residents in January 2021. This is significantly higher than case rates in Palm Springs (also in the Coachella Valley), which reported >20 cases/1,000 residents in September 2020 and > 50 cases/1,000 residents in January 2021.[37]

This pattern of increased total confirmed cases of COVID-19 in these ECV communities continued throughout the study period. In September 2020, Mecca had 455 cases increasing to 1079 cases in January 2021; North Shore had 136 cases increasing to 331 cases; Oasis had 333 cases increasing to 826 cases; and Thermal had 185 cases increasing to 440 cases [37]. An increase in deaths due to COVID-19 accompanied the increased cases. In September 2020, Mecca had 9 reported deaths increasing to 16 in January 2021; North Shore had 1 reported death and remained stable; Oasis had 5 reported deaths increasing slightly to 6; and Thermal had 0 reported deaths increasing to 4 deaths [37].

### Eligibility and Recruitment

*Promotores de salud* (community health workers) recruited community members into the study by distributing study flyers with eligibility criteria and study contact information to individuals and families in their social networks. Eligibility criteria were met if a community member: 1) was 18 years of age or older, 2) lived in the ECV and/or farm-working community along the Salton Sea, 3) self-identified as Latino/Hispanic, Latinx and/or indigenous from Latin America, and 4) spoke Spanish and/or Purepecha. Monolingual English-speaking Latinos and monolingual speakers of an indigenous dialect other than Purepecha were excluded from participation.

### Data Collection

A focus group is a group interview that allows qualitative researchers to gather collective data about a specific phenomenon of interest. This method of data collection allows participants to build on each other’s ideas,[38] providing collective (rather than individual) knowledge about the cultural and structural factors shaping COVID-19 testing and vaccination in vulnerable communities. We conducted seven virtual focus groups of six to ten people each from November to December 2020 to elicit information on shared cultural factors and structural stressors shaping COVID-19 testing behaviors and vaccine hesitancy. For nonprobability samples, 80% of themes can be identified within two to three focus groups and 90% within three to six focus groups.[39]

*Promotores* facilitated the focus groups with assistance from medical and pre-medical students. All facilitators received training on qualitative data collection and data analysis. Facilitators used a semi-structured interview guide with open-ended questions to elicit information on shared cultural beliefs and attitudes around the virus, its spread, and COVID-19 testing and future vaccination, as well as risk-reduction behaviors such as social distancing and use of face coverings. We prompted discussion about themes emerging from our conversations with community members during public health outreach and testing events, including trust in public health officials, the government, and providers/healthcare systems, as well as strategies and tools to support those with COVID-19 and increase risk-reduction behaviors and use of COVID-19 testing services. At the end of all focus groups, participants were asked to complete a socio-demographic survey, either by using a link to a Qualtrics (online) version of the survey, or by having a team member administer the survey to them via phone.

### Data Analysis

Focus groups were conducted via Zoom video conference, audio recorded, transcribed, and analyzed using template and matrix analysis, a rapid qualitative analytic technique.[40–42] *Promotores* and students participated in a 2-part training on template and matrix analysis and led data analysis with support from experts in this analytic approach. Team members read transcripts line-by-line and inserted data, including illustrative excerpts from the interviews, in the templates. Next, a matrix (focus group x domain) was created, and data from each template were inserted into the matrix. Data were analyzed and separated by the three main topics of the focus group sessions: coronavirus, COVID-19 testing, and the COVID-19 vaccine. This approach facilitated identifying themes/patterns across the seven focus groups conducted.

## Results

Seven focus groups were held from November to December 2020 with 55 total participants, 53 of whom completed the socio-demographic survey. The majority of participants were female (81 %), Hispanic or Latino (83%), age 25 to 54 (83%), and had health insurance (72%). More than half the participants represented those living in unincorporated farming communities: Thermal, Mecca, and Oasis. Over one-third (36%) of participants identified as farmworkers, suggesting a significant percent of women in the sample worked in the fields. Most (83%) participants felt affected by the coronavirus. Participants cited reduced work hours and income, inability to work or no work, childcare, and COVID-19 infection as ways they were most affected by the virus. Among survey respondents who cited childcare as a factor in how the coronavirus has affected their lives, a third theme was remote learning. Participant demographics are reported in Table 1.

As seen in Fig. 1, the intersection of SDOH and structural factors shaped perceptions around the coronavirus and COVID-19 influencing attitudes and behaviors related to the use of COVID-19 testing services and intent to vaccinate. Themes of misinformation, shaped by historically based distrust in government, public health, and medicine, as well as social factors of insecurity and fear linked to present day employment and deportation concerns emerged in participants’ discussions across focus groups. These intersection of SDOH and historically based inequalities place this Latinx population in structurally vulnerable positions, which imposes multiple forms of exclusion, discrimination, violence and emotional and physical suffering ultimately increasing the risk for COVID-19.

### Misinformation

Misinformation, i.e., incorrect or misleading information, contributes to confusion, skepticism, and/or disbelief in the COVID-19 virus and vaccination. Information about COVID-19 comes from a myriad of sources that vary from individual to individual. Participants noted some members of their communities might not have access to the internet, proficient English language skills, or knowledge about how to access reliable public health sources for information regarding COVID-19. Instead, many rely on word of mouth or social media platforms like Facebook and Snapchat. One focus group member said: “I think there should be more groups that help neighbors who do not know very well how to use the internet.” Whereas others expressed frustration with fellow community members who did utilize the internet: “But if they have Facebook, if they have Snapchat, I do not understand, that is, if they are up to date in that aspect, they must also be a little bit more up to date about the [COVID-19] tests that are offered, right?”

Lacking reliable and trustworthy information sources while having access to misinformation was common. Sources of misinformation contributed to the commonly held belief that people would get infected by going to testing sites: “A lot of people say they’re not going to get tested because they’re going to get contaminated there.” Another participant shared, “people say that by getting tested you will get the virus.”

Another major source of misinformation was the fragmented, disunited response from the previous government:

Perhaps they [community members] are confused because they see the Donald Trump administration, and he said, ‘No, no, no.’… I think people are confused about believing whether the virus is serious or not.

This lack of certainty and acknowledgement of the severity of the virus on a national level contributed to some community members not taking COVID-19 seriously. This was evident with people continuing to partake in behaviors that increase risk of spread of the virus. As will be expanded upon below, the misinformation and confusion about COVID-19 undermined trust in institutions. Mistrust is a byproduct of the misinformation and confusion around COVID-19: community members do not know what is true or what is false. One participant made a plea for “public health experts and doctors to bring information to communities, that is correct, so that people are no longer confused...”

### Lack of Trust in Institutions

Lack of confidence in government entities (e.g., the political administration, public health) due to the anti-immigrant political context played a major role in the attitudes and beliefs held by community members. Participants talked about community perceptions of the government and public health working together to harm minority groups. One participant commented: “In the beginning, really early on, when the virus was just starting, I was hearing everywhere, they said it [the spread of the virus] was political.”

Across focus groups, participants discussed the role of government in the pandemic. Some discussed community members’ fear that the government incited the virus as a form of state control: “I heard that if you get the vaccine, it’s so the government can control you.” Others shared concerns that the vaccine is harmful: “If we get vaccinated, we might get more sick, or are going to die.” Additional beliefs or “myths,” as participants referred to them, about the vaccine included: the COVID-19 vaccine was actually a way for the government to implant a microchip to control and monitor behavior, or contained another deadly virus. One participant illustrated this point: “My neighbors say ‘No’ [to getting the vaccine], because they [the government] are going to put a chip in them, or because they might put another virus [in them]. that’s what people from my community think”.

In light of the mistrust in government, participants vocalized wanting governmental leaders to get vaccinated first so they could observe the vaccine’s effects: “When the vaccine comes out [and] when government officials get vaccinated...let’s look at the reaction.” Participants did not want to be test subjects or “guinea pigs” for the vaccine, they feared a repeat of the harm caused by previous medical studies on minorities.

Lack of trust in institutions extends beyond government into hospitals and healthcare. One participant said: “I have heard that people don’t want to go to the hospital because they let you die.”

### Insecurity

Insecurity around employment and residency (e.g., fear of deportation linked to undocumented status) shaped decision making around COVID-19 testing. Many participants shared fear within their community about accessing COVID-testing services due to the possibility that a positive test result could lead to loss of employment or deportation:

There is fear that you can lose your job if you test positive for the virus. The migration situation is a barrier that makes it difficult for people to get tested, they fear that they [testing service] will share their information.

Immigration and citizenship status create barriers to COVID-19 testing services and shape ideas around anticipated vaccination. Identification and being identified as undocumented are significant concerns. Participants stated that a form of identification is the first thing requested at COVID-19 testing centers: “many people who live in [community names], they have no ID. So many people don’t get the test because they don’t have documents, and they don’t have identification.”

Misinformation and mistrust in institutions have led to increased levels of insecurity and fear among participants in our focus groups. There is fear of the government locating this immigrant population and going after them. One participant shared the following about a friend: “He’s afraid because they’re saying the government wants to wipe out the whole town.”

## Discussion

The current body of literature on the impact of COVID-19 on vulnerable populations is limited in scope, particularly in terms of studies involving Black, Indigenous, and People of Color (BIPOC), including Latinx communities, as well as studies providing in-depth qualitative insights. Our study deepens understanding of the effects of COVID-19 among vulnerable Latinx communities and establishes a baseline data understanding of the role of misinformation, lack of trust in institutions, and insecurity around employment and deportation as contributing to COVID-19 testing service and vaccination hesitation.

Our findings provide evidence of how a history of institutionalized racism and present-day anti-immigrant sentiments act as social and structural determinants of health shaping understanding of the coronavirus and COVID-19, ultimately increasing risk for contagion and disease. Within these Latinx communities, there is a persistent fear that vaccination for COVID-19 is intended to control the Latinx immigrant population in the US. As our findings highlight, community members fear vaccination will result in implantation of a microchip tracking device, female sterilization, and/or contagion. Such findings speak to lack of trust in government and public health systems.

Marginalized and vulnerable populations have valid reasons to distrust the government.[43] The history of medical experimentation on African Americans/Blacks[43] has, over centuries, contributed to mistrust and suspicion of institutions associated with authority and the production of medical knowledge, including the academic/scientific community, public health and healthcare systems, and government. Add into this dark history the anti-immigration and xenophobic attitudes promulgated by policies of exclusion, such as the public charge enacted by the former Presidential administration, that negatively affect immigrant health. [44, 45] Fear and apprehension shape ideas around COVID-19 testing and vaccination; to get tested or vaccination means trusting in public health structures and agencies. Rebuilding trust in institutions within vulnerable populations is critical to addressing social and structural determinants of health in an effort to reduce barriers to healthcare services use, specifically COVID-19 testing and vaccination.

As we found in our research, one way to (re)build trust in institutions is to engage those most vulnerable to COVID-19 (i.e., BIPOC) in decision-making around public health outreach and service delivery. In our study, *promotores* who identified as immigrant and/or indigenous Latinos engaged members of their community in COVID-19 testing and research. Additionally, attention should be focused on delivering public health information and news in a way that is clear and accessible to diverse communities, particularly underserved and marginalized communities who may not have access to mainstream media sources or broadband Internet connection. Our research encourages sharing of COVID-19 material for vulnerable Latinx communities through community and ethnic media sources such as print, radio, and television. Other approaches may include promotores talks led by promotores or other trusted members of the community such as religious leaders (e.g., priests) or medical doctors well-integrated into the community.

Limited access to evidence-base knowledge sources can perpetuate misinformation. During discussions about community perceptions in our study, one persistent myth arose that hospitals “let people die.” Throughout the pandemic, hospitals across the nation have been short on beds, ventilators, staff, and other medical resources. A major goal of public health safety measures has been to slow the spread of the coronavirus to flatten the curve, preventing surges in infection and the number of infected patients, thereby reducing the burden on healthcare systems, staff, and medical resources. [46] Yet, as research shows, the key to flattening the curve involves effective social distancing in the community.[47]

As our findings indicate, throughout the pandemic, including in the height of wave 2 of virus spread in fall and winter 2020, community members in the ECV continued to gather for celebrations and did not adhere to strict social distancing measures. Ideas such as those shared by participants in our study, could contribute to lack of trust in health care systems and public health. This finding suggests the need for stronger efforts to share information on the purpose of social distancing, what it means to flatten the curve, and what happens when severe cases of COVID-19 enter care. For instance, information about common treatments for severe cases of COVID-19, as well as survival rates for intubated patients could be widely disseminated to reduce fear and build trust in medicine and public health.

### Limitations

Study findings offer insight into the factors shaping COVID-19 testing and vaccine hesitancy among structurally vulnerable Latinx communities; however, several limitations should be considered when interpreting the findings. First, we conducted this research in November and early December 2020, prior to the emergency approval of COVID-19 vaccines. Thus, discussions about vaccine hesitancy were centered on future vaccines and the possibility of vaccination, not actual vaccines and vaccination. Second, we used a snowball sampling technique and recruited participants via *promotores*’ social networks within Latinx farm-working communities. Participant experiences may not reflect those of Latinx immigrants in farm-working communities in other regions or non-immigrant Latinx communities. Last, our team conducted focus groups in Spanish and Purépecha and excluded English-speaking Latinos and other indigenous-language speakers. Thus, our findings may not reflect the experiences of more acculturated Latinx community members.

## Conclusions

Social and structural factors increase risk for COVID-19 transmission and death. Mistrust in the government and fears of deportation due to anti-immigrant sentiments and policies may create significant barriers to utilization of COVID-19 testing services and vaccine uptake among Latinx immigrant communities across the US. Solutions must focus on addressing the social and structural determinants of health among vulnerable populations in the context of COVID-19. Acknowledging the role of structural inequities in Latinx communities and fostering trust with institutions (government, public health) in order to decrease hesitancies in COVID-19 testing and vaccination services is needed.

## Figures and Tables

**Table 1:** Demographics and characteristics of participants

	N = 53
*Demographics*	N(%)
Gender	
Female	43(81)
Male	10(19)
Ethnicity/Race
Hispanic or Latino	44(83)
Purépecha	9(17)
Age	
18 to 24	5(9)
25 to 44	21 (40)
45 to 54	23(43)
55–64	4(8)
Health insurance	
Yes	38(72)
No	15(28)
Community	
Unincorporated farm-working communities (Thermal, Mecca, Oasis)	30(56)
Indio	7(13)
Salton City	3(6)
Coachella	11(21)
Other	2(4)
Employment status	
Part-time (less than 40 hours/week)	18(34)
Full-time (more than 40 hours/week)	10(19)
Stay-at-home parent	8(15)
Student	2(4)
Unemployed	11(21)
Disabled	4(8)
Farmworker	
Yes	19(36)
No	34(64)
Have you felt affected by coronavirus?	
Yes	44(83)
No	8(15)
No Response	1(2)

* A total of 55 people participated in one of the focus groups; only 53 completed the socio-demographic survey.

## List Of Abbreviations

BIPOC: Black, Indigenous, and People of Color
CBPR: Community based participatory research
COVID-19: Coronavirus disease 2019
ECV: Eastern Coachella Valley
SARS-CoV-2: Severe acute respiratory syndrome coronavirus 2
SDOH: Social determinants of health
